# Feasibility of Fluid Responsiveness Assessment in Patients at Risk for Increased Intracranial Pressure

**DOI:** 10.3390/jcm13061786

**Published:** 2024-03-20

**Authors:** Aleksandar R. Zivkovic, Aleko Kjaev, Silvia Schönenberger, Sandro M. Krieg, Markus A. Weigand, Jan-Oliver Neumann

**Affiliations:** 1 Medical Faculty Heidelberg, Department of Anesthesiology, Heidelberg University, Im Neuenheimer Feld 420, 69120 Heidelberg, Germany; aleko.kjaev@med.uni-heidelberg.de (A.K.); markus.weigand@med.uni-heidelberg.de (M.A.W.); 2 Medical Faculty Heidelberg, Department of Neurology, Heidelberg University, Im Neuenheimer Feld 400, 69120 Heidelberg, Germany; silvia.schoenenberger@med.uni-heidelberg.de; 3 Medical Faculty Heidelberg, Department of Neurosurgery, Heidelberg University, Im Neuenheimer Feld 400, 69120 Heidelberg, Germany; sandro.krieg@med.uni-heidelberg.de

**Keywords:** intensive care unit, passive leg raise test, volume responsiveness, hemodynamics, end-expiratory occlusion maneuver, leg raising test, traumatic brain injury, fluid tolerance

## Abstract

**Background:** Effective fluid management is important for patients at risk of increased intracranial pressure (ICP). Maintaining constant cerebral perfusion represents a challenge, as both hypovolemia and fluid overload can severely impact patient outcomes. Fluid responsiveness tests, commonly used in critical care settings, are often deemed potentially hazardous for these patients due to the risk of disrupting cerebral perfusion. **Methods:** This single-center, prospective, clinical observational study enrolled 40 patients at risk for increased ICP, including those with acute brain injury. Informed consent was obtained from each participant or their legal guardians before inclusion. The study focused on the dynamics of ICP and cerebral perfusion pressure (CPP) changes during the Passive Leg Raise Test (PLRT) and the End-Expiratory Occlusion Test (EEOT). **Results:** The results demonstrated that PLRT and EEOT caused minor and transient increases in ICP, while consistently maintaining stable CPP. EEOT induced significantly lower ICP elevations, making it particularly suitable for use in high-risk situations. **Conclusions:** PLRT and EEOT can be considered feasible and safe for assessing fluid responsiveness in patients at risk for increased ICP. Notably, EEOT stands out as a preferred method for high-risk patients, offering a dependable strategy for fluid management without compromising cerebral hemodynamics.

## 1. Introduction

Acute brain injury (ABI) following major stroke, traumatic brain injury, subarachnoid or intracranial hemorrhage is a prevalent condition with significant health implications, often resulting in lasting changes in brain function and structure. It encompasses a spectrum from mild neurologic impairment to severe brain damage, characterized by a broad array of symptoms and disabilities. In neurocritical care, the primary focus is on stabilizing patients with ABI and mitigating secondary brain injury, a challenge compounded by the complexity of maintaining optimal cerebral perfusion [1,2,3].

The major challenge in neurocritical care lies in effectively balancing cerebral blood flow (CBF) and intracranial pressure (ICP) to prevent ischemic injuries and facilitate recovery in patients with acute brain injury [4]. Achieving and maintaining adequate cerebral perfusion pressure (CPP) is essential in this delicate process [5]. This involves the precise monitoring of ICP, mean arterial pressure (MAP) and possibly brain tissue oxygenation (PbtO2), as well as navigating the intricacies of fluid management. The goal is to ensure sufficient cerebral blood flow without risking cerebral edema or increased ICP, which are critical considerations in the comprehensive care of these patients [6,7].

Fluid management in neurocritical care, particularly in ABI cases, demands a meticulous approach. The objective is to maintain hemodynamic stability without causing fluid overload, which can lead to increased intracranial pressure and worsen cerebral edema [8,9,10]. Traditional guidelines often recommend using fluid balances or central venous pressure (CVP) to guide volume status with the aim of increasing cardiac output [11]. However, the effectiveness of CVP as a guide for fluid therapy in critically ill patients, including those with ABI, has been questioned in recent literature, which suggests that static indices like CVP might not accurately reflect a patient’s fluid responsiveness [12,13]. This highlights the need for a more nuanced approach to fluid management in the neurocritical care setting, considering the unique challenges posed by ABI.

The Passive Leg Raise test (PLRT) and the End-Expiratory Occlusion test (EEOT) have emerged as potential tools for more accurately assessing volume responsiveness [14,15,16,17,18]. Established in intensive care for their simplicity, cost-effectivity and low risk, these methods have not been widely adopted in neurocritical care for ABI patients due to concerns regarding exacerbating intracranial hypertension [14]. PLRT, which induces a reversible increase in preload, and EEOT, which evaluates cardiopulmonary interactions in mechanically ventilated patients, offer opportunities to optimize fluid therapy if proven safe and effective in this context.

In this study, a single-center, prospective pilot investigation, we seek to rigorously assess the safety and feasibility of employing PLRT and EEOT in the neurocritical care of ABI patients, particularly those with elevated ICP. By exploring the effects of these fluid responsiveness tests on ICP and CPP, our objective is to clarify their role and potential utility in a setting where conventional fluid management approaches may be insufficient. This research endeavors to provide empirical evidence to either validate or reconsider the current reservations about using PLRT and EEOT in managing high-risk neurocritical care patients, thus contributing to a more nuanced understanding of fluid management in ABI.

## 2. Materials and Methods

### 2.1. Study Design

The study was designed as a prospective, clinical, single-center observational study. To mitigate potential cross-effects between the PLRT and EEOT, the sequence was randomized in a 1:1 ratio. The absence of sufficient data in the existing literature precluded meaningful power analysis; thus, the sample size was set at 40 subjects. A ‘washout phase’ between the sequences was implemented to reduce carryover effects from the first test. The cutoff for test interruption was an ICP value higher than 25 mmHg [19]. Baseline measurements included ICP, CPP, systemic arterial pressure and heart rate. The PLRT, followed by a second set of measurements, was conducted as described in the existing literature [14]. The test concluded after 60–90 s or if the ICP exceeded 25 mmHg, followed by a “reassessment” measurement set. The EEOT protocol involved a 15 s end-expiratory occlusion, with measurements taken before and directly following the occlusion, and again two minutes post-conclusion [17]. Continuous ICP monitoring was performed throughout the PLRT and EEOT protocols, ensuring the consistent real-time observation and recording of ICP changes. Tests could be interrupted by the attending physician in cases of hemodynamic instability, ICP > 25 mmHg, or any adverse effects threatening patient safety. In one study case, a patient’s intracranial pressure (ICP) briefly exceeded the established threshold, peaking at 25.3 mmHg during PLRT. Despite this slight surpassing of the 25 mmHg limit, the test was continued, and the data from this case were included in the final analysis. The primary outcome of the study focused on the changes in ICP during the PLRT and EEOT, assessing the safety and physiological impacts of these maneuvers. Secondary outcomes were expanded to include variations in end-tidal CO_2_ (etCO_2_) and changes in systemic blood pressure during the procedures, providing a comprehensive evaluation of respiratory, metabolic and circulatory responses.

### 2.2. Ethical Approval and Patient Consent

This study was conducted with the approval of the Ethics Committee of Heidelberg University Hospital (S-692/2022, granted on 23 November 2022), ensuring adherence to ethics standards in line with the Declaration of Helsinki. Informed consent was obtained from all study participants or their legal guardians. The study was designed to minimize patient risk and interference with standard care; examinations conducted did not influence the duration of inpatient stay or interfere with routine therapeutic or diagnostic measures, ensuring the integrity of patient care and data confidentiality through pseudonymization.

### 2.3. Patient Selection

**Figure 1:**
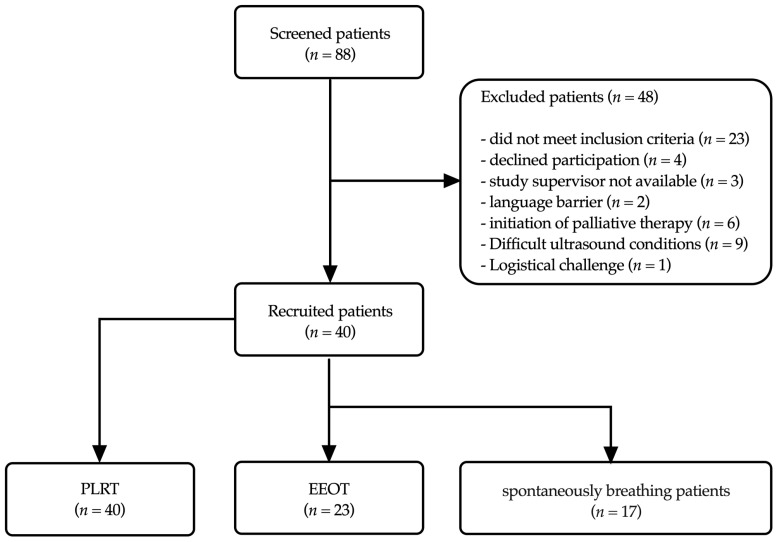
Flowchart of patient recruitment showing numbers of screened, included and excluded patients based on study criteria.

The study was conducted in the neurosurgical and neurological intensive care unit of Heidelberg University Hospital. Eligible patients were those aged 18 years or older who had suffered a traumatic brain injury, a major stroke, or intracranial or subarachnoid hemorrhage (either spontaneous or traumatic). Over a period of 10 months, from January to November 2023, in total, 40 non-consecutive patients were enrolled. The study included both spontaneously breathing and ventilated patients (Figure 1). Exclusion criteria for this study included refusal by the patient or their legal guardian, the absence of an external ventricular drainage/parenchymal probe for intracranial pressure monitoring, the absence of an arterial cannula for continuous blood pressure monitoring, pregnancy, and sustained pathologically elevated intracranial pressure.

### 2.4. Measurements and Monitoring

#### 2.4.1. ICP Monitoring Techniques

ICP and CPP values were continuously measured using either an external ventricular drain (EVD) or a parenchymal ICP probe (RAUMEDIC^®^ Neuronvent-P, Helmbrechts, Germany). EVDs are implemented primarily in patients where simultaneous intracranial pressure monitoring and cerebrospinal fluid (CSF) drainage are clinically warranted. The insertion of an EVD involves placing a catheter within one of the brain’s lateral ventricles, enabling a direct measurement of ventricular fluid pressure. This technique not only allows for accurate ICP monitoring but also provides a means to alleviate elevated ICP through controlled CSF drainage. For continuous ICP monitoring without the necessity for CSF drainage, parenchymal probes are utilized. These probes are inserted into the brain’s parenchymal tissue, offering the advantage of minimal tissue disruption and high measurement accuracy. Parenchymal probes are characterized by their robust construction and reliability in providing stable ICP readings over extended periods, making them particularly suited for long-term monitoring in diverse patient populations. ICP monitoring techniques were selected based on the specific clinical needs and therapeutic goals for individual patients, including patients’ clinical conditions, the intended monitoring duration, and the need for therapeutic CSF drainage. Data recording was facilitated by RAUMEDIC^®^ Datalogger MPR1 (RAUMEDIC^®^, Helmbrechts, Germany) and managed using RAUMEDIC Datalogger software (RAUMEDIC^®^, Helmbrechts, Germany, Ver. 1.7).

#### 2.4.2. Arterial Line Placement and Monitoring

Arterial catheters (Arterial Leadercath, Vygon^®^, Ecouen, France) were inserted for continuous blood pressure monitoring and arterial blood gas analysis. The radial or femoral artery was chosen for the placement of the arterial line, based on the patient’s condition and the clinician’s preference. This invasive method allows for the real-time, beat-to-beat monitoring of systemic arterial pressure, which is crucial for calculating CPP and managing hemodynamic stability. Monitoring of vital parameters was carried out using Dräger Infinity Delta^®^ monitor (Drägerwerk AG & Co. KgaA, Lübeck, Germany).

#### 2.4.3. Cerebral Perfusion Pressure Measurement

Cerebral perfusion pressure was calculated as the difference between the mean arterial pressure and intracranial pressure; CPP = MAP − ICP. The arterial line provided the MAP values, while ICP was measured using either an EVD or a parenchymal probe, as previously described.

#### 2.4.4. Mechanical Ventilation

For mechanically ventilated patients, the Evita V800^®^ respirator (Drägerwerk AG & Co. KgaA, Lübeck, Germany) was set to pressure control mode, with adjustments made to achieve target tidal volumes of 6–7 mL/kg to ensure adequate oxygenation. End-tidal CO_2_ (etCO_2_) monitoring of mechanically ventilated patients was conducted using Dräger CO_2_-Sensor for Evita V800, to maintain etCO_2_ within the desired range. Importantly, etCO_2_ monitoring was not performed on patients who were breathing spontaneously.

### 2.5. Protocols for the Assessment of Fluid Responsiveness

#### 2.5.1. Passive Leg Raise Test (PLRT)

The PLRT was conducted as follows: Initially, the patient was positioned in bed with the upper body elevated between 30 and 45 degrees, followed by the baseline measurement of hemodynamic parameters (ICP, CPP, systolic, mean, diastolic blood pressure, heart rate and end-tidal CO_2_). The patient was then passively repositioned to lay their upper body flat at 0 degrees and elevate their legs to 30–45 degrees. Subsequently, the hemodynamic measurements were repeated (test measurement). The test concluded after 60–90 s, by returning the patient to the starting position, followed by a final set of hemodynamic measurements (reassessment measurement), provided the upper ICP limit of 25 mmHg was not exceeded.

#### 2.5.2. End-Expiratory Occlusion Test (EEOT)

The EEOT was performed in mechanically ventilated patients. Ventilation parameters (tidal volume, respiratory rate, ventilation mode, inspiration-to-expiration ratio, PEEP, FiO_2_ and etCO_2_) were documented and kept unaltered during the test. The patient’s position with the upper body elevated between 30 and 45 degrees remained unchanged throughout the test. After an initial (baseline) measurement of hemodynamic parameters, a 15 s breath hold was performed on a respirator (Evita V800^®^, Drägerwerk AG & Co. KgaA, Lübeck, Germany), as described by Monnet et al. [17]. Ventilation was resumed with the pre-set parameters, and the second (test) measurement of the hemodynamic parameters was performed. A third (reassessment) measurement was taken five minutes after completing the EEOT.

#### 2.5.3. Reason for Disparity in Measurement Numbers between PLRT and EEOT

The PLRT protocol included both ventilated and spontaneously breathing patients (*n* = 40), whereas EEOT measurements could only be performed for ventilated patients (*n* = 23). Consequently, the number of EEOT measurements was lower than that of PLRT measurements.

#### 2.5.4. Sedation during Tests

No additional sedation was required for either spontaneously breathing or mechanically ventilated patients undergoing the PLRT and EEOT protocols.

### 2.6. Statistical Analysis

Data normality was assessed using the Kolmogorov–Smirnov and Shapiro–Wilk tests. Results were reported as medians with interquartile ranges. The Friedman and Mann–Whitney tests were used to determine statistical significance, set at a *p*-value < 0.05. Analyses were conducted using GraphPad Prism 10 (GraphPad Software, San Diego, CA, USA).

## 3. Results

This study included 40 patients with acute brain injury. Patient demographics and clinical characteristics are detailed in Table 1.

The protocol involved the PLRT and EEOT, conducted in a randomized sequence. Initial assessments were performed at the earliest feasible timepoint within 24 h of hospital admission (acute stage), followed by secondary assessments conducted at least 24 h later (subacute stage). The median interval between these stages was 2 days (range: 1–4 days).

The principal objective of the study was to evaluate the safety and impact of fluid responsiveness tests on cerebral perfusion by measuring variations in intracranial and cerebral perfusion pressure.

**Figure 2:**
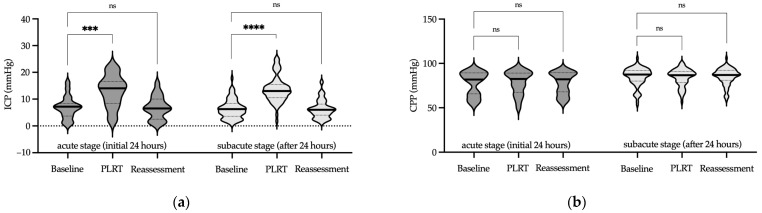
ICP and CPP measurements during PLRT. Violin plots illustrate ICP (**a**) and CPP (**b**) at the acute (dark gray) and subacute (light gray) stages of ABI during the PLRT protocol. This protocol involves baseline, test, and reassessment phases, where a notable transient increase in ICP is observed, in contrast to the stable CPP throughout the procedure. Median values are marked by solid lines, while quartiles are represented by dashed lines. Significance levels are denoted as follows: *** *p* < 0.001, **** *p* < 0.0001, ns—not significant (Friedman test). Abbreviations: ICP—intracranial pressure; CPP—cerebral perfusion pressure; PLRT—Passive Leg Raise Test.

Focusing first on the PLRT protocol, we investigated ICP changes across the acute and subacute stages of brain injury. In the acute stage, PLRT resulted in a significant transient increase in ICP, which normalized by the reassessment phase (*p* < 0.001 in the test phase and *p* = 0.66 in the reassessment phase, Friedman test, Figure 2a, Table S1). In the subacute stage, similar transient elevation in ICP was observed, with the median value remaining within physiological limits (*p* < 0.0001 in the test phase and *p* = 0.99 in the reassessment phase, Friedman test, Figure 2a, Table S1).

Regarding CPP during the PLRT protocol, the acute stage revealed stable levels throughout the assessment phases (*p* = 0.29 in the test phase and *p* = 0.66 in the reassessment phase, Friedman test, Figure 2b, Table S1). In the subacute stage, CPP remained unchanged during the protocol (*p* = 0.18 in the test phase and *p* = 0.99 in the reassessment phase, Friedman test, Figure 2b, Table S1). Notably, in the acute phase, a few isolated cases exhibited slightly lower CPP values, although these did not reach statistical significance. These instances of marginally reduced CPP were carefully monitored, and no clinical consequences were observed. Importantly, this trend was not evident in the subacute phase, where CPP was consistently maintained above the critical threshold of 60 mmHg throughout the PLRT protocol (Figure 2b, Table S1).

**Figure 3:**
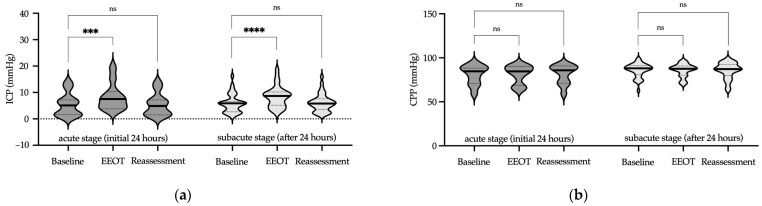
ICP and CPP measurements during the EEOT. Violin plots present ICP (**a**) and CPP (**b**) during the acute (dark gray) and subacute (light gray) phases of ABI under the EEOT protocol. This protocol, comprising baseline, test, and reassessment stages, shows a transient rise in ICP, while CPP displays consistent stability. Median values are highlighted with bold lines, and interquartile ranges are shown as dotted lines. Significance levels: *** *p* < 0.001, **** *p* < 0.0001, ns—not significant (Friedman test). Abbreviations: ICP—intracranial pressure; CPP—cerebral perfusion pressure; EEOT—end-expiratory occlusion test.

Next, we examined the impact of the EEOT protocol on ICP and CPP during both stages of ABI. During the acute stage, the EEOT led to a significant increase in ICP, which returned to the baseline in the reassessment phase (*p* < 0.001 in the test phase and *p* = 0.99 in the reassessment phase, Friedman test, Figure 3a, Table S1). In the subacute stage, the EEOT induced a transient, yet notable increase in ICP that returned to the baseline during reassessment (*p* < 0.0001 in the test phase and *p* = 0.99 in the reassessment phase, Friedman test, Figure 3a, Table S1).

Cerebral perfusion pressure during the EEOT protocol remained stable in the acute stage, as evidenced by consistent measurements in both the test and reassessment phases (*p* = 0.99 for each phase, Friedman test, Figure 3b, Table S1). In the subacute stage, this stability of CPP was similarly maintained, indicating that the EEOT protocol had no significant effect on cerebral perfusion (*p* = 0.99 in the test phase and *p* = 0.95 in the reassessment phase, Friedman test, Figure 3b, Table S1). Thus, the results indicate the effective preservation of CPP during the EEOT protocol, throughout both the acute and subacute stages of ABI.

**Figure 4:**
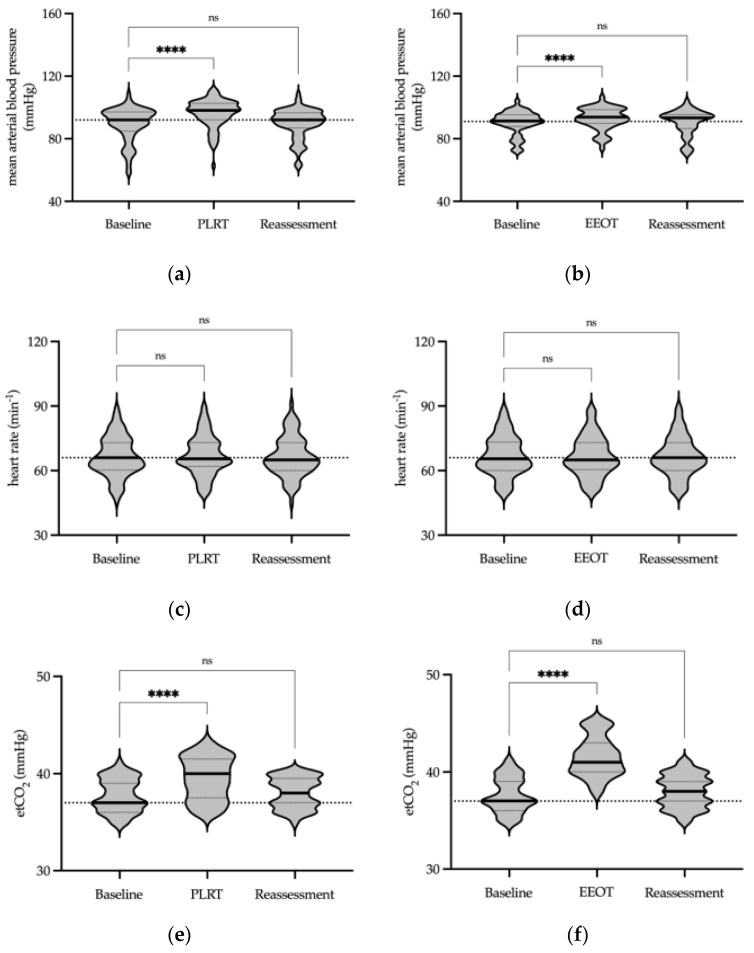
Hemodynamic and respiratory parameters in the PLRT and EEOT. Violin plots display transient increases in mean arterial pressure during the PLRT (**a**) and EEOT (**b**). Heart rate stability is shown throughout both tests (**c**,**d**). End-tidal CO_2_ concentrations exhibit significant increases during the PLRT (**e**) and EEOT (**f**), yet remain within physiological ranges. Test measurements are categorized into baseline, test, and reassessment phases. Median values are indicated by thick lines, and quartiles by dotted lines. Significance levels: **** *p* < 0.0001, ns—not significant (Friedman test). Abbreviations: etCO_2_—end-tidal carbon dioxide; PLRT—passive leg raise test; EEOT—end-expiratory occlusion test.

We subsequently evaluated the hemodynamic and respiratory parameters during the PLRT and EEOT protocols. A notable transient increase in mean arterial pressure was observed during the PLRT (*p* < 0.0001 in the test phase and *p* = 0.99 in the reassessment phase, Friedman test, Figure 4a, Table S1) as well as during the EEOT (*p* < 0.0001 in the test phase and *p* = 0.87 in the reassessment phase, Friedman test, Figure 4b, Table S1). However, heart rate consistently remained stable throughout both the PLRT and EEOT protocols, with no significant fluctuations (PLRT: *p* = 0.08 in the test phase and *p* = 0.50 in the reassessment phase; EEOT: *p* = 0.50 in the test phase and *p* = 0.75 in the reassessment phase, Friedman test, Figure 4c,d, Table S1).

Disruption in carbon dioxide maintenance, caused by respiratory and circulatory alterations, might affect hemodynamic and metabolic equilibrium in these patients. We therefore tested whether or not fluid assessment protocols might cause alterations in end-tidal carbon dioxide. A transient and brief, yet significant, increase in etCO_2_ concentration was observed during the PLRT (*p* < 0.0001 in the test phase and *p* = 0.21 in the reassessment phase, Friedman test, Figure 4e, Table S1) and during the EEOT assessment protocol (*p* < 0.0001 in the test phase and *p* = 0.29 in the reassessment phase, Friedman test, Figure 4f, Table S1). Throughout both assessment protocols, etCO_2_ levels were maintained within physiological levels, indicating that the fluid responsiveness tests did not lead to prolonged disruptions in the carbon dioxide balance.

**Figure 5:**
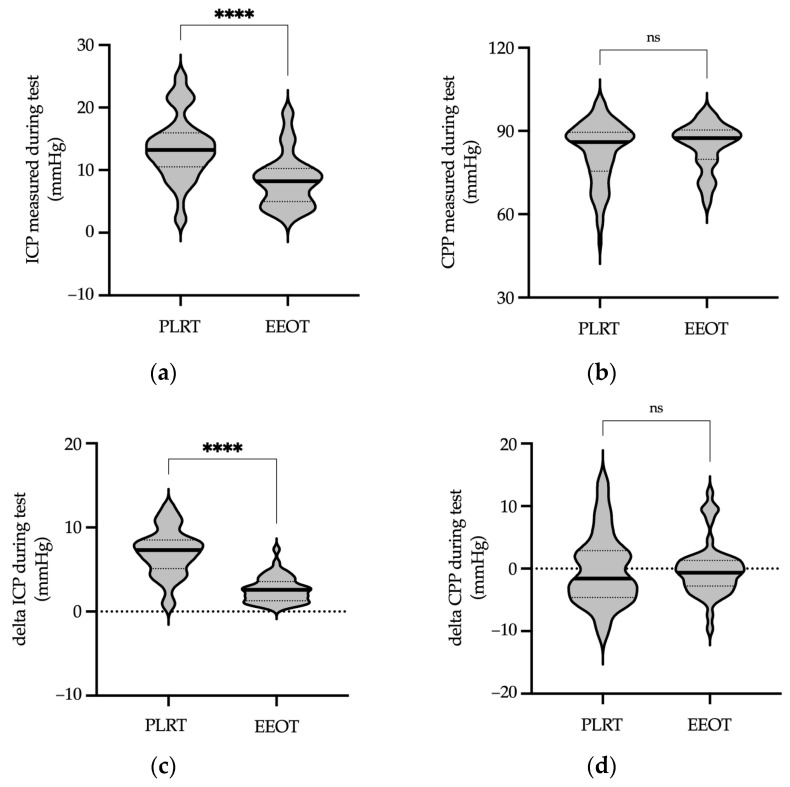

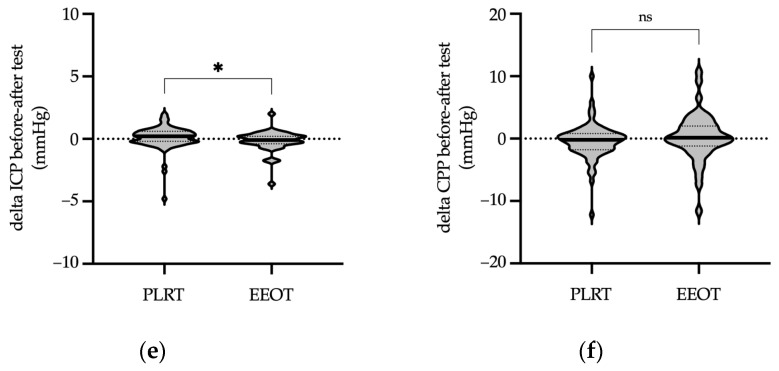
Comparative analysis of cerebral perfusion parameters between PLRT and EEOT protocols. Panel (**a**) displays the maximal measured ICP value during each protocol, with significantly higher ICP observed in the PLRT compared with the EEOT. Panel (**b**) presents the maximal measured CPP values, showing consistent measurements across both protocols with no significant differences. Panel (**c**) showcases the calculated maximal difference in ICP (delta ICP) between the peak and baseline values during the test, compared between the PLRT and EEOT protocols. This analysis reveals a significantly higher delta ICP in the PLRT compared to with the EEOT. Panel (**d**) displays the calculated maximal difference in CPP (delta CPP) under similar conditions, indicating no significant difference in delta CPP between the two protocols, suggesting comparable CPP changes in the PLRT and EEOT. Panel (**e**) presents the analysis of changes in ICP before and after the PLRT and EEOT, comparing the delta ICP (difference between pre-test and post-test ICP). The analysis indicates a statistically significant difference in delta ICPs between the protocols, with a greater change observed in the PLRT. Panel (**f**) performs the same analysis for CPP, assessing the delta CPP (difference between pre-test and post-test CPP). The results show no significant difference in delta CPPs between the PLRT and EEOT. Importantly, these results indicate that there were no lasting changes in either ICP or CPP following the completion of both protocols. Median values are marked by thick lines, and quartiles by dotted lines. Significance levels: * *p* < 0.05, **** *p* < 0.0001, ns—not significant (Mann–Whitney test). Abbreviations: ICP—intracranial pressure; CPP—cerebral perfusion pressure; PLRT—passive leg raise test; EEOT—end-expiratory occlusion test.

In our subsequent analysis, we focused on determining differences between the two fluid responsiveness assessment protocols. Specifically, ICP was significantly higher during the PLRT compared with the EEOT (*p* < 0.0001, Mann–Whitney test, Figure 5a, Table S1). In contrast, CPP remained consistent regardless of the protocol used, showing no significant changes in either the PLRT or EEOT (*p* = 0.47, Mann–Whitney test, Figure 5b, Table S1).

The extent of ICP increase, indicated by the maximal difference observed during the test (delta ICP during test), was more substantial in the PLRT than in the EEOT (*p* < 0.0001, Mann–Whitney test, Figure 5c, Table S1). Conversely, the maximal difference in CPP during the test (delta CPP during test) remained constant across both protocols (*p* = 0.72, Mann–Whitney test, Figure 5d, Table S1).

Furthermore, a comparison of ICP levels before and after the fluid responsibility assessment tests (delta ICP before–after test) was performed to evaluate potential permanent changes in cerebral perfusion following the performed protocols. This analysis revealed a statistically significant difference between the protocols (*p* < 0.05, Mann–Whitney test, Figure 5e, Table S1). However, it is important to note that no permanent changes in ICP or CPP were detected following the completion of both the PLRT and EEOT protocols in patients with acute brain injury (Figure 5e,f, Table S1).

**Figure 6:**
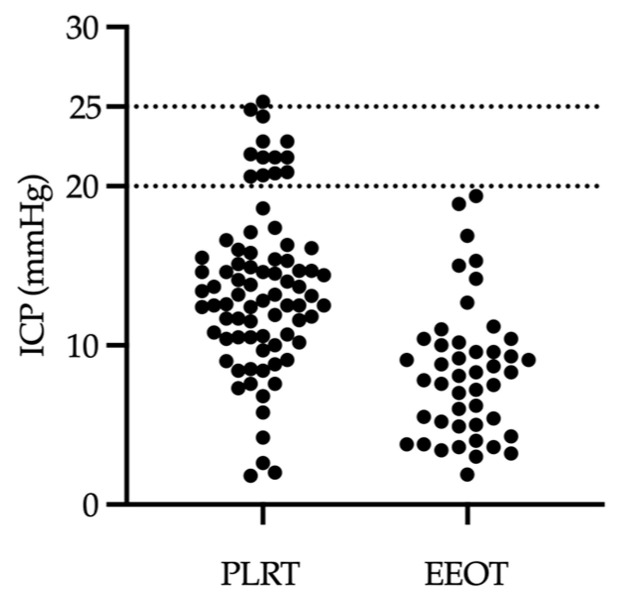
Individual peak ICP measurements in PLRT and EEOT protocols. The scatter plot displays the peak ICP values recorded for each patient during the PLRT and EEOT protocols. It highlights the instances where ICP exceeded 20 mmHg during the PLRT, in contrast to a complete absence of such instances in the EEOT, effectively illustrating the differential impact of the two protocols on peak ICP values. Abbreviations: ICP—intracranial pressure; PLRT—passive leg raise test; EEOT—end-expiratory occlusion test.

Finally, we addressed the primary focus of our study: to determine if hemodynamic alterations during fluid assessment protocols could lead to a significant increase in measured ICP. In a case-by-case analysis of the 80 ICP measurements taken during the PLRT, we found that 13 instances, or 16.25% of the measurements, showed ICP values exceeding 20 mmHg. Importantly, none of these readings surpassed 25 mmHg, with the exception of a single case, where there was a brief spike to 25.3 mmHg (as detailed in the study design in the Materials and Methods section). In contrast, none of the ICP readings recorded during the EEOT exceeded the level of 20 mmHg (Figure 6). These findings suggest that the fluid assessment protocols described here are feasible for use in patients with acute brain injury.

## 4. Discussion

This study offers valuable insights into the feasibility of using fluid responsiveness assessments in neurocritical care, particularly for patients with acute brain injury who are at risk of elevated intracranial pressure. Our findings suggest that both the Passive Leg Raise Test and the End-Expiratory Occlusion Test can be safely conducted in such high-risk scenarios.

The data indicate that during the PLRT, ICP mostly remained below the critical threshold of 25 mmHg. These results are significant as they counter the prevailing concerns about using the PLRT in patients with acute intracranial conditions. Occurrences of ICP reaching the 20–25 mmHg range were rare and short-lived, aligning with previous research that reported the safety of the PLRT in a small patient group with ABI [19]. This reinforces the perspective that dynamic assessments like the PLRT and EEOT may provide a more accurate reflection of fluid responsiveness than can static parameters such as CVP, which has been shown to have limited predictive value in critical care settings [20,21]. Incorporating these insights, it becomes evident that the reliance on CVP as a static parameter for fluid management in patients with acute brain injury is fraught with limitations. The evolving evidence advocates for a shift toward dynamic indices and a more individualized approach to fluid therapy, considering the unique hemodynamic complexities of each patient [13].

Remarkably, cerebral perfusion pressure remained stable throughout both the PLRT and EEOT procedures. Despite fluctuations in ICP, cerebral perfusion pressure remained stable throughout both the PLRT and EEOT, indicating that these tests do not compromise cerebral perfusion. The observed stability in CPP is essential, as maintaining adequate cerebral blood flow is of utmost importance for preventing secondary brain injuries in patients with ABI [10,22]. This finding is in line with the understanding that fluid management plays a vital role in the management of acute brain injury patients, especially considering the potential risks of hyperhydration during initial therapy [23].

In the context of the EEOT, we observed an even more reassuring safety profile. This test showed no significant rise in ICP over 20 mmHg, suggesting that the EEOT might be a preferable method for fluid responsiveness assessment in acute-phase, high-risk cases. The stability of CPP during the EEOT adds further credence to its safety and applicability in neurocritical care settings.

Another vital aspect of our study is the monitoring of end-tidal CO_2_. It is well documented that increased CO_2_ levels can lead to elevated cerebral blood flow and, consequently, increased ICP due to CO_2_-induced vasoregulation [24]. Our observations of mild increases in end-tidal CO_2_ during the tests, though not substantial, highlight the importance of careful CO_2_ monitoring. This finding underscores the need for rigorous end-tidal CO_2_ control to prevent inadvertent cerebral hemodynamic changes that could negatively impact patients with ABI.

In conducting our study, we expanded upon the groundwork laid by Bauer et al. in the field of fluid management for patients with acute brain injury [19]. Aligning with their initial findings, our investigation confirms the practicality of Passive Leg Raising (PLR) in the neurointensive care setting. However, our approach diverges in several aspects. By incorporating a substantially larger patient cohort, we enhanced the statistical reliability of our results and broadened the scope of applicability to a more diverse ABI population. Additionally, the introduction of two distinct protocols in our study permits a more detailed comparative analysis, offering finer insights into the fluid dynamics in ABI. A particularly salient feature of our study is the adoption of a 24 h observation window for the acute stage of ABI, as opposed to the 72 h period employed in Bauer et al.’s research. This modification allowed us to better observe and differentiate changes that occurred during the initial phase of ABI, offering a better understanding of the pathologic processes in this crucial early stage.

Our research also sheds light on the broader challenges of fluid management in neurocritical care. Patients with brain injury are especially susceptible to imbalanced volume management due to alterations in their intravascular volume and neuroendocrine disturbances [9,25,26]. Fluid overload, which must be carefully avoided, can precipitate intracranial hypertension and disrupt stable cerebral perfusion, thereby elevating the risk of delayed cerebral ischemia [27]. The results of our study suggest that both the PLRT and EEOT can be safely incorporated into the fluid management strategies in neurocritical care, offering an enhanced approach to maintaining normovolemia.

Our findings, emphasizing the safety of the PLRT and EEOT in neurocritical care, particularly in ABI patients, must be contextualized within the broader research landscape, including the recent study by Messina et al. [28] Their investigation, focusing on the effects of the PLRT on intracranial pressure and cerebral autoregulation, concluded that while the PLRT did not critically elevate ICP, it might have impaired cerebral autoregulation. This led the authors to advise caution in using the PLRT for ABI patients with stable ICP. The differing interpretation by Messina et al., largely attributed to the extensive use of advanced multimodal neuromonitoring, contrasts our approach and the findings of Bauer et al. [19]. Nevertheless, the insights provided by Messina et al. are invaluable, adding depth to the ongoing discussion about fluid responsiveness assessments in neurocritical care. Despite these differences, all studies contribute to understanding the complex aspects of fluid management in ABI patients.

Our study’s contribution includes a larger patient cohort, which adds statistical weight to our results. It is the combination of our findings with those from Bauer and Messina et al. that highlights the complexities of managing fluid responsiveness in ABI, with each study offering a unique perspective.

In conclusion, the collective findings from these studies emphasize the complex nature of managing fluid responsiveness in ABI. By integrating diverse methodologies and interpretations, we collectively move closer to optimizing fluid management strategies in neurocritical care, ultimately aiming to improve patient outcomes in these challenging clinical situations.

Despite its contributions, our study has limitations that warrant consideration. The single-center setting and observational design may limit the generalizability of our findings, as they might not reflect the diversity of practices and patient populations across different institutions and regions. The sample size, while the largest published in this context so far, remains relatively small, potentially limiting the statistical power of the study. Next, direct measurements of brain oxygenation, perfusion flow, brain saturation, and central venous pressure were not conducted due to technical and logistical constraints. Furthermore, a critical aspect pertains to the use of parenchymal ICP probes for intracranial pressure monitoring. While these devices offer the advantage of minimal invasiveness and high accuracy, they are not without potential measurement drifts over time. Indeed, the literature suggests that parenchymal probes can experience a minimal drift, typically ranging from −0.13 to 0.11 mmHg per day [29]. Although this drift is relatively minor, it is essential to consider its potential impact on prolonged ICP monitoring. In the context of our study, which primarily focused on the change in ICP, rather than absolute values, we postulate that the influence of this drift on our findings is likely minimal. However, it is important to acknowledge that any measurement drift, however slight, could introduce a degree of variability into the data. Finally, the scope of our research did not extend to a direct comparison of the efficacy of the PLRT and EEOT in determining actual fluid responsiveness. These limitations suggest a direction for future research, which should encompass a broader scope, including direct comparisons of these tests and measurements of brain-specific parameters.

## 5. Conclusions

In conclusion, both the Passive Leg Raise Test and the End-Expiratory occlusion test have demonstrated their safety for fluid responsiveness assessment sin patients susceptible to increased intracranial pressure. The tests, including the PLRT, generally result in only minor and brief elevations in intracerebral pressure. Notably, the continuity of cerebral perfusion pressure across these procedures reaffirms their efficacy and safety. Of particular significance is that the EEOT emerges as a valuable method in critical early stage scenarios, offering a dependable approach for fluid management in neurocritical settings.

## Figures and Tables

**Table 1:** Patient demographics clinical characteristics.

**Patient characteristics**	
Age (years)	65 (56–73)
Gender (male/female)	27/13 (67.5%/32.5%)
Body mass index (kg/m^2^)	25.5 (23.8–28.2)
**Referring diagnosis**	
Subarachnoid hemorrhage (SAH)	12 (30%)
- Non-traumatic SAH	9 (22.5%)
- Traumatic SAH	3 (7.5%)
Intracerebral hemorrhage (ICH)	9 (22.5%)
- Non-traumatic ICH	6 (15%)
- Traumatic ICH	3 (7.5%)
Subarachnoid and intracerebral hemorrhage	5 (12.5%)
Subarachnoid hemorrhage and subdural hematoma	3 (7.5%)
Intraventricular hemorrhage	3 (7.5%)
Secondary acute hydrocephalus *	5 (12.5%)
Secondary (postoperative) hemorrhage	2 (5%)
Traumatic subdural hematoma	1 (2.5%)
**Comorbidities**	
Arterial hypertension	16 (40%)
Atrial fibrillation	6 (15%)
Diabetes mellitus type 2	3 (7.5%)
COPD	4 (10%)
Chronic kidney disease	1 (2.5%)
Coronary heart disease	4 (10%)
Smoking	2 (5%)
**Clinical course and treatment**	
GCS	7 (3–14)
Days in ICU	15 (11–20)
Antibiotic therapy	30 (75%)
Catecholamine therapy	22 (55%)
Pneumonia	18 (45%)
**Respiratory parameters** (**of ventilated patients**)	
Tidal volume (ml/kg)	7 (6–7)
PEEP (cmH_2_O)	5 (5–5)
Driving pressure (cmH_2_O)	8.5 (7–10)
Respiratory rate (min^−1^)	14 (14–15)
Dynamic compliance (ml/cmH_2_O)	64 (55–72)
Fraction of inspired oxygen	0.35 (0.35–0.40)

* Hydrocephalus was attributed to acute occlusion resulting from edema, which was secondary to cerebral cavernoma in 2 patients, intraventricular cyst in 1 patient, cerebellar dermoid in 1 patient, and shunt infection in 1 patient. Values are medians with interquartiles unless specified otherwise. Abbreviations: COPD—chronic obstructive pulmonary disease; GCS—Glasgow Coma Scale; ICU—intensive care unit; PEEP—positive end-expiratory pressure.

## Data Availability

The patient data that support the findings of this study are not publicly available due to privacy and ethical restrictions. However, the data are available from the corresponding author upon reasonable request and with the provision of a proposal outlining the purpose of data use. All requests will be subjected to an approval process to ensure the proposed use of the data is in line with ethical guidelines and legal requirements related to patient data confidentiality and consent.

## Supplementary Materials

The following supporting information can be downloaded at: https://www.mdpi.com/article/10.3390/jcm13061786/s1: Table S1: A comprehensive summary of the study results.
