# Supplementary material for: Feasibility of Fluid Responsiveness Assessment in Patients at Risk for Increased Intracranial Pressure
Source: J Clin Med. 2024 Mar 20;13(6):1786. doi: 10.3390/jcm13061786 (PMC10970886; doi:10.3390/jcm13061786)
Supplement: Supplementary file 1 [file jcm-13-01786-s001.zip › jcm-2879252-supplementary.pdf]

Parameter	Baseline	Test	Reassessment
	**PLR test (acute stage)**		
ICP (mmHg)	7.2 (3.7 – 8.3)	14.0 (8.4 – 16.6)	6.5 (2.5 – 10.0)
CPP (mmHg)	81.8 (65.7 – 89.3)	82.4 (67.4 – 89.1)	82.0 (67.9 – 89.5)
	**PLR test (subacute stage)**		
ICP (mmHg)	6.3 (3.6 – 8.4)	13.0 (10.5 – 15.4)	6.0 (4.0 – 8.1)
CPP (mmHg)	87.4 (80.0 – 91.9)	86.7 (78.5 – 91.0)	86.9 (81.0 – 92.2)
	**EEO test (acute stage)**		
ICP (mmHg)	5.1 (1.7 – 7.2)	7.5 (3.8 – 10.4)	4.9 (1.5 – 7.2)
CPP (mmHg)	84.7 (70.5 – 88.3)	84.5 (68.6 – 90.1)	85.8 (71.2 – 90.8)
	**EEO test (subacute stage)**		
ICP (mmHg)	5.9 (2.7 – 6.6)	8.7 (5.2 – 10.2)	5.8 (3.6 – 7.6)
CPP (mmHg)	88.0 (80.9 – 91.0)	87.6 (80.3 – 90.7)	87.2 (80.0 – 92.6)
	**PLR test**		
ICP (mmHg)	6.4 (3.9 – 8.4)	13.2 (10.5 – 16.0)	6.05 (3.4 – 8.5)
ICP change score (mmHg)		7.3 (5.1 – 8.5)	-0.2 (-0.6 – 0.2)
ICP difference (mmHg)			0.2 (-0.2 – 0.6)
CPP (mmHg)	86.6 (78.3 – 91.1)	86.0 (75.5 – 89.5)	86.5 (77.0 – 90.7)
CPP change score (mmHg)		-1.6 (-4.6 – 2.9)	0.2 (-0.8 – 1.8)
CPP difference (mmHg)			-0.2 (-1.8 – 0.8)
Systolic blood pressure (mmHg)	131 (126 – 136)	145 (136 – 148)	132 (126 – 137)
Mean blood pressure (mmHg)	92 (85 – 97)	98 (92 – 103)	92 (87 – 97)
Diastolic blood pressure (mmHg)	72 (65 – 77)	75 (71 – 80)	72 (67 – 76)
Heart rate (min^{-1})	66 (60 – 73)	66 (62 – 73)	65 (60 – 73)
$etCO_2$ (mmHg)	37 (36 – 39)	40 (38 – 42)	38 (37 – 40)
	**EEO test**		
ICP (mmHg)	5.8 (2.5 – 7.0)	8.2 (5.0 – 10.0)	5.8 (2.6 – 7.3)
ICP change score (mmHg)		2.6 (1.3 – 3.6)	0.1 (-0.2 – 0.4)
ICP difference (mmHg)			-0.1 (-0.4 – 0.2)
CPP (mmHg)	88 (80 – 90)	87 (80 – 90)	87 (78 – 92)
CPP change score (mmHg)		-0.65 (-2.8 – 1.3)	-0.15 (-2.0 – 1.2)
CPP difference (mmHg)			0.2 (-1.2 – 2.0)
Systolic blood pressure (mmHg)	131 (128 – 138)	137 (132 – 142)	132 (128 – 137)
Mean blood pressure (mmHg)	91 (88 – 95)	94 (90 – 99)	93 (87 – 95)
Diastolic blood pressure (mmHg)	72 (67 – 84)	73 (68 – 77)	72 (66 – 77)
Heart rate (min^{-1})	66 (60 – 73)	65 (61 – 73)	66 (60 – 73)
$etCO_2$ (mmHg)	37 (36 – 39)	41 (40 – 43)	38 (37 – 39)

Table S1: A comprehensive summary of the study results
